# Association between Alzheimer's disease pathologic products and age and a pathologic product-based diagnostic model for Alzheimer's disease

**DOI:** 10.3389/fnagi.2024.1513930

**Published:** 2024-12-19

**Authors:** Weizhe Zhen, Yu Wang, Hongjun Zhen, Weihe Zhang, Wen Shao, Yu Sun, Yanan Qiao, Shuhong Jia, Zhi Zhou, Yuye Wang, Leian Chen, Jiali Zhang, Dantao Peng

**Affiliations:** ^1^Graduate School, Beijing University of Chinese Medicine, Beijing, China; ^2^Department of Neurology, China-Japan Friendship Hospital, Beijing, China; ^3^Department of Orthopedics, Handan Chinese Medicine Hospital, Handan, Hebei, China

**Keywords:** Alzheimer's disease, pathologic product, machine learning, age, diagnostic model, linear regression

## Abstract

**Background:**

Alzheimer's disease (AD) has a major negative impact on people's quality of life, life, and health. More research is needed to determine the relationship between age and the pathologic products associated with AD. Meanwhile, the construction of an early diagnostic model of AD, which is mainly characterized by pathological products, is very important for the diagnosis and treatment of AD.

**Method:**

We collected clinical study data from September 2005 to August 2024 from the Alzheimer's Disease Neuroimaging Initiative (ADNI) database. Using correlation analysis method like cor function, we analyzed the pathology products (t-Tau, p-Tau, and Aβ proteins), age, gender, and Minimum Mental State Examination (MMSE) scores in the ADNI data. Next, we investigated the relationship between pathologic products and age in the AD and non-AD groups using linear regression. Ultimately, we used these features to build a diagnostic model for AD.

**Results:**

A total of 1,255 individuals were included in the study (mean [SD] age, 73.27 [7.26] years; 691male [55.1%]; 564 female [44.9%]). The results of the correlation analysis showed that the correlations between pathologic products and age were, in descending order, Tau (Corr=0.75), p-Tau (Corr=0.71), and Aβ (Corr=0.54). In the AD group, t-Tau protein showed a tendency to decrease with age, but it was not statistically significant. p-Tau protein levels similarly decreased with age and its decrease was statistically significant. In contrast to Tau protein, in the AD group, Aβ levels increased progressively with age. In the non-AD group, the trend of pathologic product levels with age was consistently opposite to that of the AD group. We finally screened the optimal AD diagnostic model (AUC=0.959) based on the results of correlation analysis and by using the Xgboost algorithm and SVM algorithm.

**Conclusion:**

In a novel finding, we observed that Tau protein and Aβ had opposite trends with age in both the AD and non-AD groups. The linear regression curves of the AD and non-AD groups had completely opposite trends. Through a machine learning approach, we constructed an AD diagnostic model with excellent performance based on the selected features.

## 1 Introduction

Alzheimer's disease (AD) is a neurodegenerative disease that seriously jeopardizes human health and affects patients' quality of life (2023, 2024). It is the number one cause of dementia and mainly affects the middle-aged and elderly population (2022; Scheltens et al., 2021). Before developing Alzheimer's disease, patients will go through the stages of subjective memory complaints (SMC), mild cognitive decline and so on. How to diagnose Alzheimer's disease more accurately and distinguish it from the preclinical stage of Alzheimer's disease as well as normal people has been a hot topic of research. Previous researchers have attempted to differentiate AD using biomarkers, imaging, and some behavioral-based kinesiology tests, among others, with biomarker research undoubtedly receiving the most attention (Bai et al., 2021; Winchester et al., 2023; Küçükali et al., 2023; Yang et al., 2020). A large number of biomarkers have been detected in blood, cerebrospinal fluid tests, etc., which have a good ability to differentiate between patients with AD (Izzo et al., 2021; Kumari et al., 2022). The researchers even spent a great deal of time studying longitudinal changes in these biomarkers, monitoring changes in their levels throughout the course of the onset of Alzheimer's disease (Jia et al., 2024; Yakoub et al., 2023). And with the continuous advancement of histologic research techniques, more and more biomarkers are being discovered in a higher throughput manner. Our team has previously uncovered a very large number of AD biomarkers through both blood and urine testing methods, using histology-related techniques, and has built an AD diagnostic model based on them (Wang et al., 2023a,b).

β-amyloid (Aβ) and Tau proteins are the focus of biomarker research as recognized markers of AD pathology. Many of both protein families have been found to be closely associated with the onset and progression of AD (Ferrari-Souza et al., 2022; Ashton et al., 2021; Horie et al., 2023). Previous studies have focused on the particular significance of these two pathologic products in the molecular mechanisms underlying the developmental process of Alzheimer's disease (Zhang H. et al., 2021; Busche and Hyman, 2020). In contrast, the association between these two pathologic products and age in the preclinical and onset stages of AD has lacked elucidation in large-sample studies (Stern et al., 2023).

In terms of research on diagnostic models for AD, there are many studies that use biomarkers as features, and not a few of them incorporate Aβ and Tau protein (Ferreiro et al., 2023). However, in previous studies, Aβ and Tau protein were hardly used as core features, and the inclusion of other biomarkers mixed the significance of the two in modeling. At the same time, previous studies also suffered from the shortcomings of using algorithms mainly focusing on regression algorithms, a single type of algorithm and a lack of sample size (Hammond et al., 2020; Gao et al., 2023). We used classification algorithms with excellent performance in this study to construct and train an AD diagnostic model using Aβ and Tau protein as the core features.

## 2 Methods

### 2.1 Design

The Alzheimer's Disease Neuroimaging Initiative (ADNI) database^1^ provided the data used in this investigation, which were gathered between September 2005 and August 2024. Established in 2003, the ADNI program is a research endeavor with the goal of examining the course of Alzheimer's disease and its preclinical phases through the use of MRI, PET, biomarkers, and clinical and neuropsychological examinations. All participating institutions' Institutional Review Boards have given their approval for the ADNI trial. All participants, or their authorized representatives, have given written informed permission to ADNI in compliance with the Declaration of Helsinki.

### 2.2 Participants

After undergoing a battery of cognitive functioning tests, each individual was assigned to one of four groups: AD, mild cognitive impairment (MCI), SMC, or control (CN). The Mini-Mental State Examination (MMSE) scores for AD were 20–26, while for CN, SMC, and MCI, they were 24–30. For CN, MCI, and AD, the Clinical Dementia Rating (CDR) was 0.5, 0.5, and ≥0.5. For the various ADNI cohorts, the enrollment processes and inclusion criteria were generally the same. Previous descriptions have been made of the specific enrollment processes and inclusion criteria for the various diagnostic categories of the ADNI cohort (Petersen et al., 2010). You could find the ADNI database protocol^2^ with detailed inclusion and exclusion requirements. In order to evaluate cognitive function, we used the MMSE.

### 2.3 Biomarker collection and analysis

CSF Tau protein and Aβ protein data from the ADNI database were used in our study. Methods of CSF collection and biomarker measurement have been previously reported (Hampel et al., 2010; Shaw et al., 2009). The ADNI database did not report outliers. However, biomarker assays have detection intervals. The upper limit of detection for Aβ is 1,700 pg/mL and the lower limit is 200 pg/mL. The upper limit of detection for t-Tau is 1,300 pg/mL and the lower limit is 80 pg/mL. The upper limit of detection for p-Tau is 120 pg/mL and the lower limit is 8 pg/mL. The detections that exceeded the detection interval the most accounted for < 15% of the total data, and only a very small number of the other detections exceeded the detection interval. For data exceeding the detection range of Tau or Aβ proteins, we took half of the lower detection limit value to replace data below the lower detection limit, and used the upper detection limit value to replace data above the upper detection limit.

### 2.4 Model constructing and training

We employed two machine learning algorithms, support vector machine (SVM) and extreme gradient boosting (XGBoost), to build AD diagnostic models. Cross-validation is used to evaluate the performance of machine learning models as well as for hyperparameter tuning. Ten-fold cross-validation was applied to the training and validation sets in order to reduce overfitting and enhance the model's functionality. We tuned the hyperparameters based on the results of the ten-fold cross-validation to get the best performing model. The best model for this study was determined by looking at the Receiver Operating Characteristic (ROC) curve and selecting the model with the highest area under the curve (AUC).

### 2.5 Statistical analysis

R software (version 4.3.1) and IBM SPSS Statistics for Windows version 27.0 were used to conduct all statistical tests. We employed nonparametric tests to compare the Non-AD (include CN, SMC, EMCI, and LMCI) and AD groups for variables like total-Tau (t-Tau), phosphorylated tau (p-Tau), Aβ, age, and MMSE that did not match the requirements of analysis of variance (ANOVA). The chi-square test was used for statistical analysis of counts like gender. The threshold for a difference to be deemed statistically significant was *p* < 0.05. Since the data are derived from public databases, the occurrence of missing values is often unavoidable. In this study, the proportion of missing values to the total data has been well over 50%. In order to minimize the error in the study, we used direct culling of missing values in the data instead of using interpolation. In contrast, this is the optimal way to ensure data integrity and accuracy.

One popular technique for determining how closely variables correlate with one another is correlation analysis. To determine the correlation coefficient and compute the correlation of variables, we utilize the cor function found in R-4.3.1. The correlation between the variables is higher the closer the correlation coefficient's absolute value is to 1.

In several contexts, the relationship between diseased products and age was estimated using linear regression analysis. Initially, we examined the relationship between pathogenic products (include Aβ, t-Tau protein, and p-Tau protein) and age in the AD and non-AD groups. We also investigated the relationship between p-Tau/t-Tau and Aβ/t-Tau and age using linear regression in an effort to better understand the relationship between these pathogenic products and age.

## 3 Results

### 3.1 Demographic and clinical characteristics of patients

A total of 1,255 individuals were included in the study (mean [SD] age, 73.27 [7.26] years; 691 male [55.1%]; 564 female [44.9%]). Six factors in all were examined: pathogenic products (Aβ, t-Tau, and p-Tau), age, sex, and MMSE scores. The average score for the MMSE test was 26.96 [3.18], the average score for Aβ was 966.19 [458.57], the average score for t-Tau was 290.57 [136.04], and the average score for p-Tau was 27.96 [14.91] for every subject. There were statistically significant differences in every attribute between the groups (Table 1).

**Table 1:** Baseline demographics and clinical characteristics.

**Characteristics**	**Total (1,255)**	**Non-AD (1,022)**	**AD (233)**	***P* value**
Age (years)	73.27	72.94	74.71	< 0.01
**Gender**				
Male (%)	55.1	54.2	58.8	0.204
Female (%)	44.9	45.8	41.2	0.204
MMSE	26.96	27.88	22.94	< 0.01
Aβ (pg/mL)	966.19	1,034.97	664.48	< 0.01
t-Tau (pg/mL)	290.57	272.79	368.51	< 0.01
p-Tau (pg/mL)	27.96	26.01	36.51	< 0.01

Aβ, β-amyloid; t-Tau, total-Tau; p-Tau, phosphorylated tau; MMSE, Minimum Mental State Examination.

### 3.2 Correlation analysis between variables

**Figure 1:**
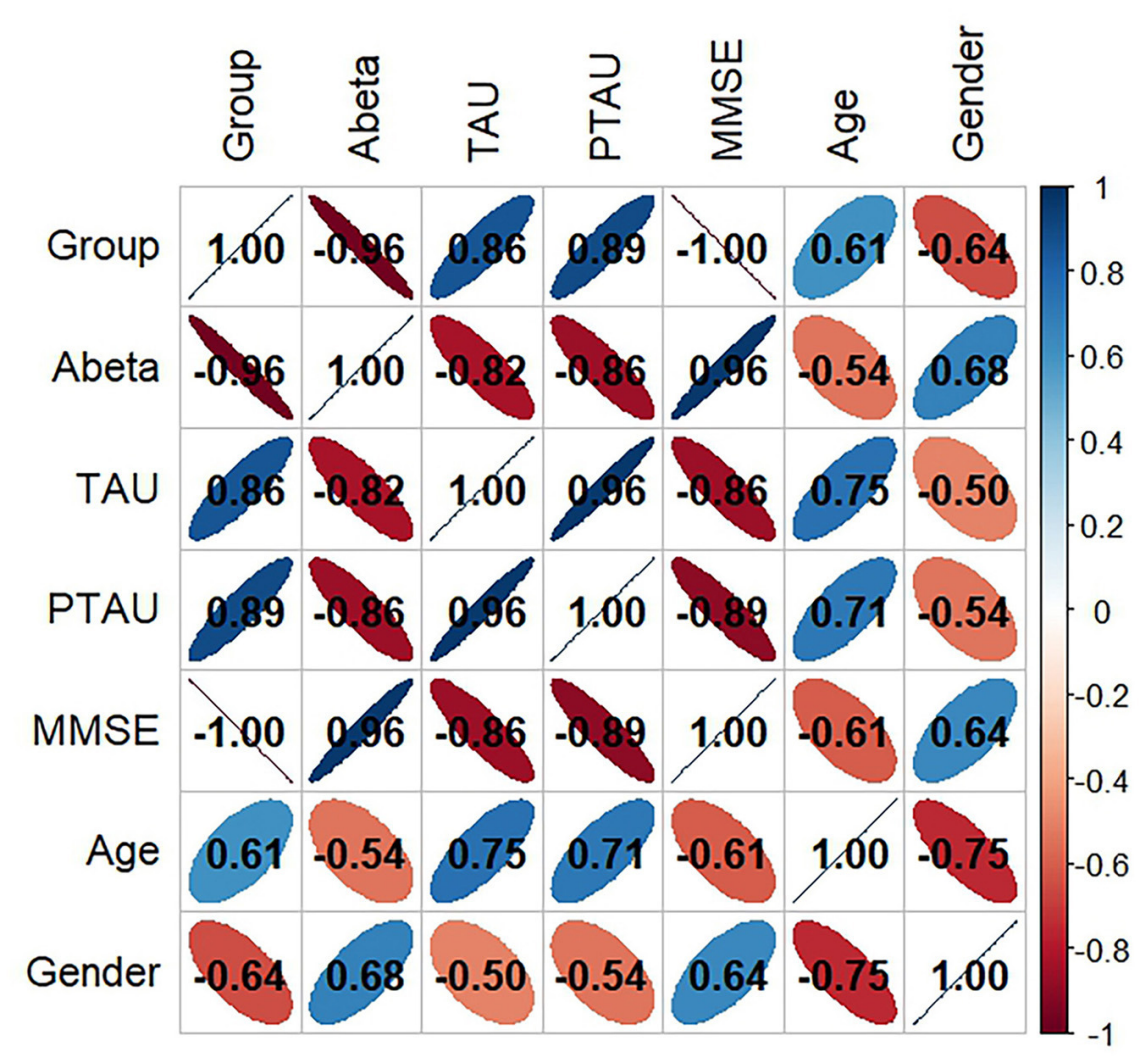
Variable correlation analysis of all features. Aβ, β-amyloid; t-Tau, total-Tau; p-Tau, phosphorylated tau; MMSE, Minimum Mental State Examination.

Figure 1 displays the findings of the six factors' correlation study with disease type. where the Corr values are between −1.0 and 1.0; the higher the correlation between the variables, the closer the Corr value's absolute value is to 1. Conversely, a correlation is less the closer it is to 0. The pathogenic products and age had the following associations, in decreasing order of absolute Corr values: t-Tau (0.75), p-Tau (0.71), and Aβ (0.54).

### 3.3 Association of Tau proteins with age

**Figure 2:**
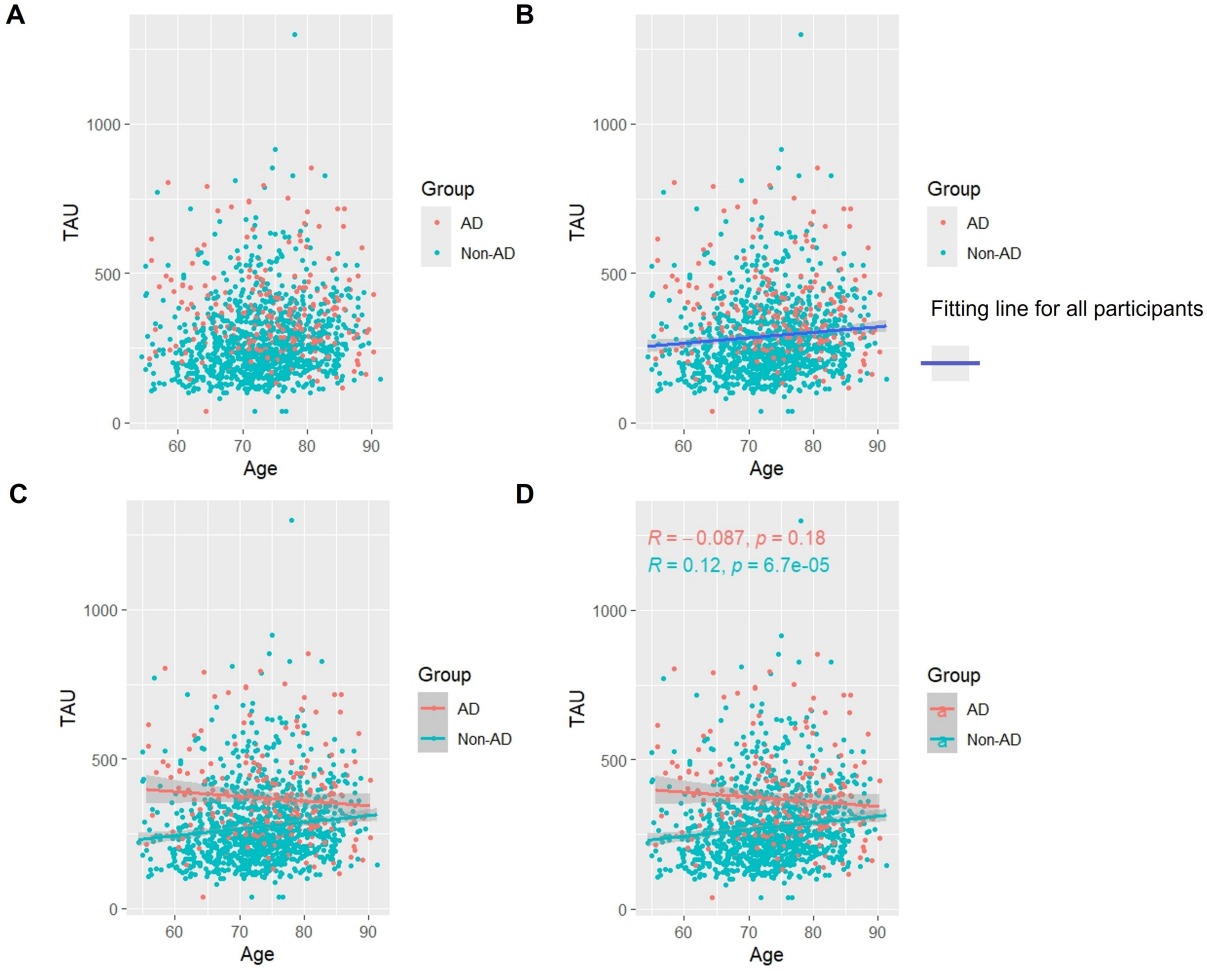
Association between t-Tau and age in AD and non-AD groups. The cyan dots and lines represent the samples and fitted lines for the non-AD group, respectively. Red dots and lines represent samples and fitted lines for the AD group, respectively. Scatterplot of the association between t-Tau and age **(A)** and Fitted curves for all participants **(B)**. Association between t-Tau and age in the AD and non-AD groups **(C)** and The p-value and R-value for each of the two groups **(D)**.

As Figure 2 shows, when we studied all subjects, we found that overall the level of Tau protein increased with age (Figures 2A, B). Interestingly, in the AD group, Tau protein appeared to slowly decrease with age, although this decrease was not statistically significant (*p* > 0.05). In the Non-AD group, the longitudinal rise in Tau protein was similarly associated with a lateral increase in age (*p* < 0.05) (Figures 2C, D).

### 3.4 Association of p-Tau protein with age

**Figure 3:**
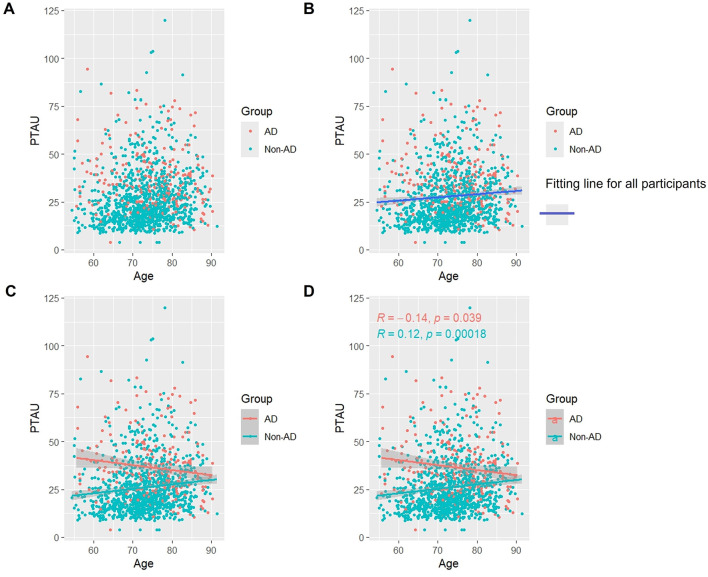
Association between p-Tau and age in AD and non-AD groups. The cyan dots and lines represent the samples and fitted lines for the non-AD group, respectively. Red dots and lines represent samples and fitted lines for the AD group, respectively. Scatterplot of the association between p-Tau and age **(A)** and Fitted curves for all participants **(B)**. Association between p-Tau and age in the AD and non-AD groups **(C)** and the *p*-value and R-value for each of the two groups **(D)**.

As Figure 3 shows, when we studied all subjects, we found that, overall, the levels of p-Tau protein increased with age (Figures 3A, B). Like t-Tau protein, in the AD group, p-Tau protein appeared to slowly decrease with age. However, the difference was that the decrease in p-Tau protein was statistically significant (p < 0.05). In the non-AD group, the longitudinal rise in p-Tau protein was similarly associated with a lateral increase in age (p < 0.05) (Figures 3C, D).

### 3.5 Association of p-Tau/t-Tau with age

**Figure 4:**
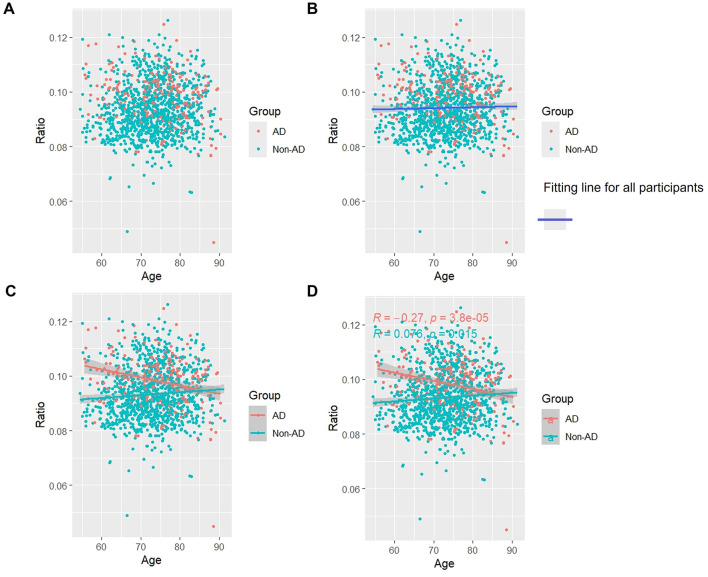
Association between p-Tau/t-Tau and age in AD and non-AD groups. The cyan dots and lines represent the samples and fitted lines for the non-AD group, respectively. Red dots and lines represent samples and fitted lines for the AD group, respectively. Scatterplot of the association between p-Tau/t-Tau and age **(A)** and Fitted curves for all participants **(B)**. Association between p-Tau/t-Tau and age in the AD and non-AD groups **(C)** and the *p*-value and R-value for each of the two groups **(D)**.

Overall, as seen in Figures 4A, B, there was a slight but steady tendency for p-Tau/t-Tau levels to rise with aging. p-Tau/t-Tau significantly decreased in the AD group as age increased, and this decline was statistically distinct (*p* < 0.05). On the other hand, p-Tau/t-Tau increased with age (*p* < 0.05) in the non-AD group (Figures 4C, D).

### 3.6 Association of Aβ with age

**Figure 5:**
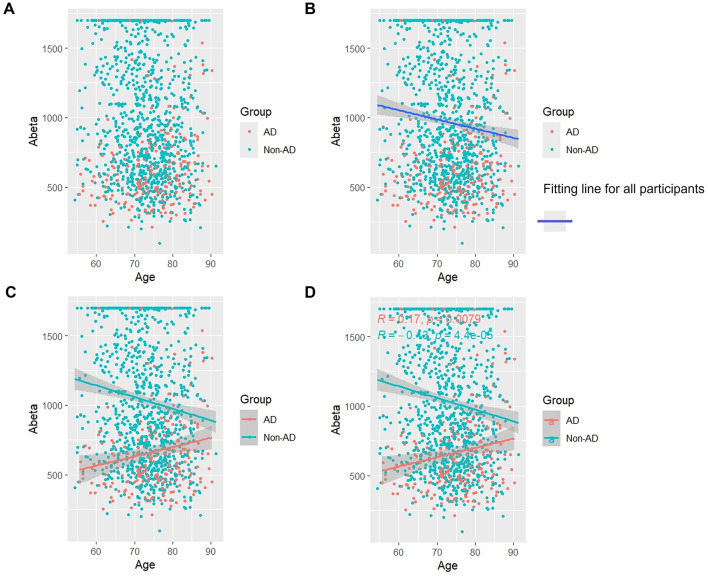
Association between Aβ and age in AD and non-AD groups. The cyan dots and lines represent the samples and fitted lines for the non-AD group, respectively. Red dots and lines represent samples and fitted lines for the AD group, respectively. Scatterplot of the association between Aβ and age **(A)** and Fitted curves for all participants **(B)**. Association between Aβ and age in the AD and non-AD groups **(C)** and the *p*-value and R-value for each of the two groups **(D)**.

Overall, as Figure 5 illustrates, Aβ levels steadily declined with age, which was different from t-Tau and p-Tau (Figures 5A, B). Aβ levels significantly increased with age in the AD group, which was likewise in contrast to t-Tau and p-Tau (*p* < 0.05). Conversely, Aβ levels in the non-AD group dropped with age (*p* < 0.05) (Figures 5C, D).

### 3.7 Association of Aβ/t-Tau with age

**Figure 6:**
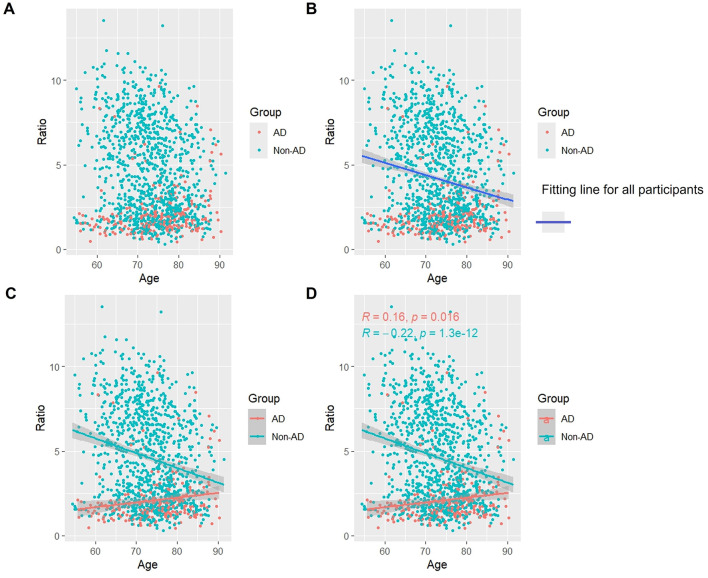
Association between Aβ/t-Tau and age in AD and non-AD groups. The cyan dots and lines represent the samples and fitted lines for the non-AD group, respectively. Red dots and lines represent samples and fitted lines for the AD group, respectively. Scatterplot of the association between Aβ/t-Tau and age **(A)** and Fitted curves for all participants **(B)**. Association between Aβ/t-Tau and age in the AD and non-AD groups **(C)** and the *p*-value and R-value for each of the two groups **(D)**.

As shown in Figure 6, as a whole, Aβ/t-Tau levels gradually decreased with age, which is consistent with the change of Aβ with age (Figures 6A, B). In the AD group, Aβ/t-Tau levels increased significantly with age (*p* < 0.05). While in the non-AD group, Aβ/t-Tau instead decreased with age (p < 0.05) (Figures 6C, D).

### 3.8 Construction and optimization of AD diagnostic models

**Figure 7:**
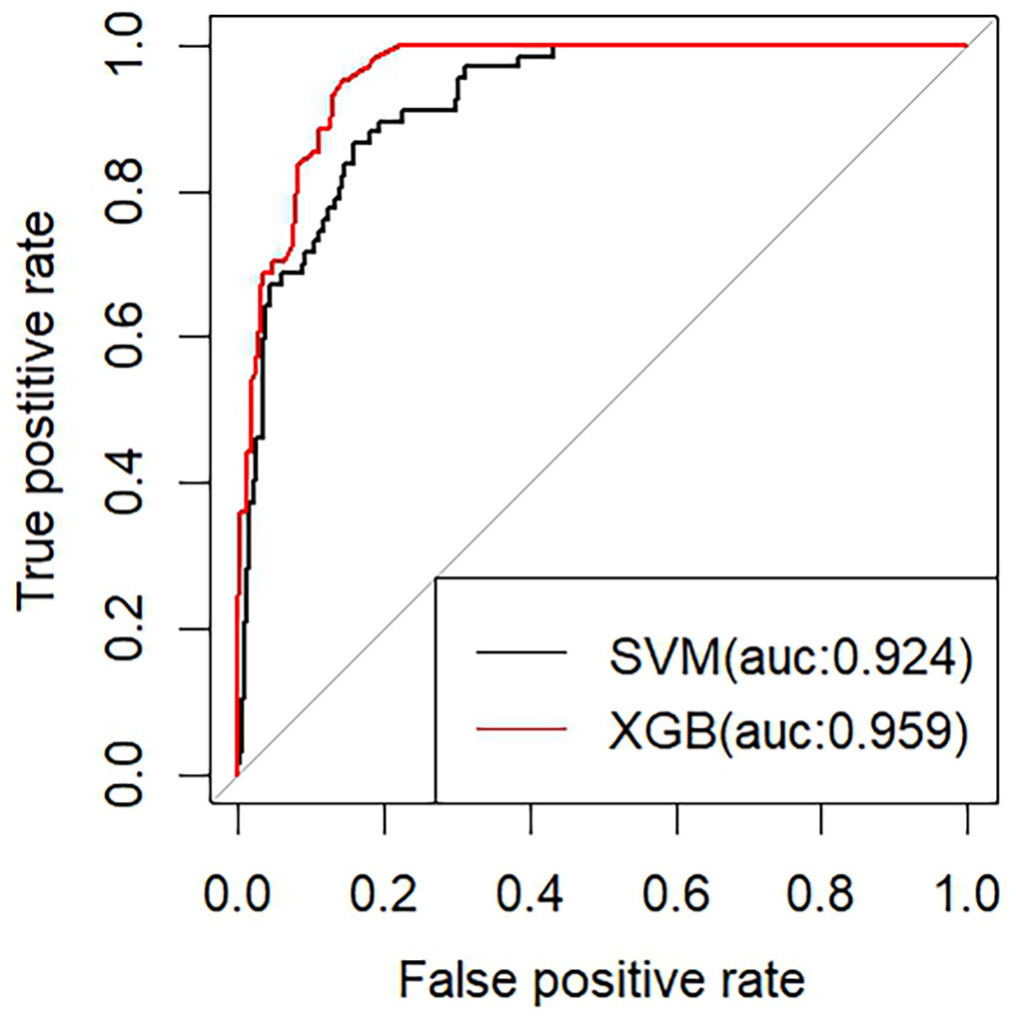
Diagnostic model of AD with pathology products as core features.

We built two machine learning models for diagnosing AD using the XGBoost classifier and the SVM classifier, respectively, based on the six previously mentioned features. To avoid overfitting, we further enhanced the model performance via ten-fold cross-validation. Among them, the classifier model based on the XGBoost algorithm has superior performance (AUC of 0.959), accuracy of 0.69, specificity of 0.86, and sensitivity of 0.95. The classifier model based on the support vector machine (SVM) algorithm has an AUC of 0.924, accuracy of 0.90, sensitivity of 0.96, and Specificity of 0.66 (Figure 7).

## 4 Discussion

Our study systematically analyzed the relationship between changes in intracranial t-Tau protein, p-Tau protein, and Aβ protein levels and age in AD patients and Non-AD population through data mining and analysis of the ADNI database. The results of the study were very interesting. We found that for both t-Tau protein and Aβ protein, the trends with age were diametrically opposed in AD patients and Non-AD populations. For both t-Tau protein and p-Tau protein, the levels of these pathogens progressively decreased the older the AD patient was. The difference, however, was that t-Tau protein showed a decreasing trend but was not statistically different compared to the statistically significant decrease in p-Tau protein. As for Aβ protein, the older the age of AD patients, the Aβ level was continuously increasing. In contrast, Aβ levels in the Non-AD population gradually declined with age. Previous studies on the correlation between these pathologic products and patient age are lacking and not clearly recognized or elaborated. However, longitudinal changes in these pathologic products over time at the individual level have been examined in previous studies, which is different from our observation of the association between pathologic products and age at the population level (Barthélemy et al., 2020). Additionally, it is not clear that some studies have focused on changes in the levels of pathologic products long before the onset of Alzheimer's disease, and have not examined changes after the onset of the disease (Jia et al., 2024). There are also studies that do not distinguish between studies of AD patients and Non-AD groups (Cogswell et al., 2024).

One of the main reasons we chose Aβ and Tau proteins for our study is that it has a very important impact in the course and mechanisms of AD (Pang et al., 2022; Sadleir and Vassar, 2023). Abnormal aggregation of Aβ and Tau proteins is an important pathogenesis and pathological hallmark of AD. Questions about how the two are produced and how their levels change during disease progression have been an important issue affecting our understanding of AD, as well as a focus and difficulty in research. Previous findings suggest that δ-secretase cleaved Tau proteins may stimulate Aβ production by upregulating STAT1-BACE1 signaling in AD patients (Zhang Z. et al., 2021). This is a rather important finding. It not only reveals the molecular regulatory process between the two pathologic products. More importantly, it suggests to us that the molecular regulatory process that exists between the two may allow the levels of these pathology products to be dynamically regulated during the course of the disease. This is likely to be an important mechanism for the pathogenesis and disease progression of AD. Not only that, Aβ pathology may induce changes in soluble tau release and phosphorylation (Mattsson-Carlgren et al., 2020). These findings show that the relationship between Aβ and t-Tau and p-Tau is bi-directionally regulated and mutually restrained. Fluctuations in the levels of the two pathogens are influenced by each other. This also gives the ratio of the two pathologic products more value for clinical studies. In turn, abnormal aggregation of the two could ultimately drive the disease progression by leading to loss of synapses, affecting synaptic function and thus disrupting memory formation (Li et al., 2018). While the roles of Aβ and Tau in the pathogenesis of AD continue to be elucidated, researchers are monitoring changes in their levels, further exploring their potential as biomarkers of the disease, and even screening for other AD biomarkers with good predictive ability based on them (Chiu et al., 2021; Boza-Serrano et al., 2022). As the technology associated with biomedical engineering continues to advance, more assays have been developed for the detection of Aβ and Tau. In addition to furthering our understanding of the pathologic processes of AD, we are discovering more pathologic processes associated with the aggregation of these pathogens. These are also one of the hotspots and directions for future research (Pichet Binette et al., 2021).

We constructed and trained a machine learning model using pathology products such as Aβ and Tau proteins as core features. In terms of the model's performance, its ability to distinguish between AD and non-AD is excellent. Previous studies have rarely focused only on the contribution of Tau and Aβ proteins to constructing AD diagnostic models, often incorporating some other biomarkers (Gaetani et al., 2021; Ficiarà et al., 2021; Franciotti et al., 2023; Khan et al., 2024). These studies have their own innovations and strengths, but inevitably have some shortcomings. The shortcomings mainly lie in the lack of performance of the diagnostic model, the small sample size, the excessive number of incorporated features, and so on. The performance of the AD diagnostic model constructed in our study is relatively superior. The relative singularity of the incorporated features can highlight more the importance of the pathology products in the model construction. Our choice of algorithms for machine learning that performs well in dealing with classification problems is an important guarantee of the sophistication of our study (Li J. et al., 2022; Yi et al., 2023; Binder et al., 2022). The Xgboost algorithm has excellent performance in dealing with classification problems. It is characterized by its ability to handle large volumes of data while maintaining accuracy and predictive performance over other classification algorithms (Yue et al., 2022; Li Q. et al., 2022). Compared to XGBoost, the SVM algorithm is slightly less accurate and less predictive. However, in some special problems, SVM has advantages that other algorithms do not have, such as when dealing with nonlinearly differentiable data and when dealing with high-dimensional data (Huang et al., 2018; Ding et al., 2022).

Undeniably, biomarker-based machine learning diagnostic models for AD are still the most dominant research (Shah et al., 2023; Kononikhin et al., 2022). The biomarkers involved in these studies include not only the pathology products that we generally recognize, but also some lipids, proteins and so on that are closely related to the pathogenesis of AD as screened by new research methods (Wang et al., 2022). The great progress in molecular biology research has also led to the expansion of the scope of clinical biomarkers (Krokidis et al., 2023). Their potential for clinical application will be enhanced if the cost of the assay can be reduced. In addition to the traditional work related to the construction of biomarker-based AD diagnostic models, more AD-related ancillary test results have been included in the study. Common neurological examinations in the clinic, such as electroencephalography, are used to construct AD diagnostic models, which also have good diagnostic performance (Parreño Torres et al., 2023). In addition to common examination means, more and more medical devices provide more dimensional examination results. As a new type of model, AD diagnostic model based on eye movement and language has a good prospect for clinical application due to its noninvasive and easy-to-operate characteristics (Jang et al., 2021). Overall, research efforts in AD machine learning diagnostic modeling have produced a large number of models with very good performance and potential for clinical applications. However, unfortunately, more comprehensive AD diagnostic models covering multidimensional markers have not yet been developed.

Inevitably, there are shortcomings in our study. In the section examining the question of the association between pathologic products and age, we had planned to compare all preclinical stages of AD separately. However, upon attempting this, we found that this would make the presentation and interpretation of the results extraordinarily difficult, and the selection of a control group of AD patients presented considerable difficulties. In terms of AD model construction and training, we were hampered by the fact that public databases do not categorize the Aβ and Tau protein families in great detail. Other than that, we mainly chose the Xgboost algorithm and SVM algorithm, which have excellent performance in solving classification problems. We have also tried other algorithms and will try to use more algorithms to try to build AD diagnostic models in the future (Zhang et al., 2023). In addition to this, our model construction has not been externally validated due to lack of data from other database sources. However, because the data provided by the ADNI database is collected from multiple centers and has a large sample size, its accuracy and reliability for real-world application will be guaranteed. In the meantime, we are currently in the process of clinically collecting additional data to complete external validation.

## Data Availability

The original contributions presented in the study are included in the article/supplementary material, further inquiries can be directed to the corresponding author.

## Footnotes

^1^ http://adni.loni.usc.edu

^2^ https://adni.loni.usc.edu/help-faqs/adni-documentation/
